# Infection with *Helicobacter pylori* may predispose to atherosclerosis: role of inflammation and thickening of intima-media of carotid arteries

**DOI:** 10.3389/fphar.2023.1285754

**Published:** 2023-10-13

**Authors:** Karl Aramouni, Roland K. Assaf, Maria Azar, Karen Jabbour, Abdullah Shaito, Amirhossein Sahebkar, Assaad A. Eid, Manfredi Rizzo, Ali H. Eid

**Affiliations:** ^1^ Faculty of Medicine, American University of Beirut, Beirut, Lebanon; ^2^ Biomedical Research Center, Department of Biomedical Sciences at College of Health Sciences, College of Medicine, Qatar University, Doha, Qatar; ^3^ Biotechnology Research Center, Applied Biomedical Research Center, Department of Biotechnology, School of Pharmacy, Pharmaceutical Technology Institute, Mashhad University of Medical Sciences, Mashhad, Iran; ^4^ Department of Health Promotion, Mother and Child Care, Internal Medicine and Medical Specialties, University of Palermo, Palermo, Italy; ^5^ Department of Basic Medical Sciences, College of Medicine, QU Health, Qatar University, Doha, Qatar

**Keywords:** cardiovascular disease, oxidative stress, extra-gastric disease, CagA, vitamin B12 deficiency

## Abstract

Atherosclerosis is a major instigator of cardiovascular disease (CVD) and a main cause of global morbidity and mortality. The high prevalence of CVD calls for urgent attention to possible preventive measures in order to curb its incidence. Traditional risk factors of atherosclerosis, like age, smoking, diabetes mellitus, dyslipidemia, hypertension and chronic inflammation, are under extensive investigation. However, these only account for around 50% of the etiology of atherosclerosis, mandating a search for different or overlooked risk factors. In this regard, chronic infections, by *Helicobacter pylori* for instance, are a primary candidate. *H. pylori* colonizes the gut and contributes to several gastrointestinal diseases, but, recently, the potential involvement of this bacterium in extra-gastric diseases including CVD has been under the spotlight. Indeed, *H. pylori* infection appears to stimulate foam cell formation as well as chronic immune responses that could upregulate key inflammatory mediators including cytokines, C-reactive protein, and lipoproteins. These factors are involved in the thickening of intima-media of carotid arteries (CIMT), a hallmark of atherosclerosis. Interestingly, *H. pylori* infection was found to increase (CIMT), which along with other evidence, could implicate *H. pylori* in the pathogenesis of atherosclerosis. Nevertheless, the involvement of *H. pylori* in CVD and atherosclerosis remains controversial as several studies report no connection between *H. pylori* and atherosclerosis. This review examines and critically discusses the evidence that argues for a potential role of this bacterium in atherogenesis. However, additional basic and clinical research studies are warranted to convincingly establish the association between *H. pylori* and atherosclerosis.

## 1 Introduction

Atherosclerosis is precipitated by the pathological formation of plaques within blood vessels. These plaques are instigated by endothelium injury followed by infiltration of immune cells and proliferation of vascular smooth muscle cells. Thickening of the intima and hardening of the vessels then ensue, thus narrowing the vascular lumen which progresses into partial or complete obstruction of blood vessels. Atherosclerosis is a dominant cause and risk factor of cardiovascular disease (CVD), a major instigator of global mortality (Herrington et al., 2016; Libby et al., 2019). Some of the specific risk factors include age, smoking, diabetes mellitus, dyslipidemia, hypertension, and chronic inflammation (Siasos et al., 2014; Spence and Pilote, 2015; Aarabi et al., 2018; Libby et al., 2018; Yao et al., 2019; Lechner et al., 2020). These can account only for around 50% of the incidence of atherosclerosis (Herrington et al., 2016; Libby et al., 2019), making investigation into other risk factors of incidence a hot and rather attractive research area. In recent years, some attention has been given to newly identified potential atherosclerosis risk factors like chronic infections (Pothineni et al., 2017). Possible chronic infectious agents that have been reportedly linked to atherosclerosis include the infamous bacterium *Helicobacter pylori* (Karbasi-Afshar et al., 2015).

*H. pylori* is a gram-negative, microaerophilic, spiral-shaped bacterium. It is a key microorganism of the human microbiome and a colonizer of the gut of at least half of the world’s population (Eusebi et al., 2014; Mentis et al., 2015; Burucoa and Axon, 2017; Leja et al., 2019). In 1994, this bacterium was categorized as a human class I carcinogen by the World Health Organization (Wang, 2014). Not only that, but *H. pylori* infection is the major cause of several gastric diseases including acute and chronic active gastritis, chronic atrophic gastritis, peptic ulcer, gastric adenocarcinoma, and type B low-grade mucosa-associated lymphoid tissue lymphoma (Wang et al., 2014; Wroblewski and Peek, 2016; Chang et al., 2018; de Brito et al., 2019; Sugano, 2019; Ford et al., 2020). Importantly, *H. pylori* infection has been linked to more than 50 extra-gastrointestinal manifestations in a variety of medical specializations such as dermatology, endocrinology, hematology, and cardiology, among others (Campuzano-Maya, 2014; He et al., 2022). In particular, insulin resistance (Polyzos et al., 2011), liver disorders (Waluga et al., 2015), ventilator-associated pneumonia (Dadashi and Hosseinzadeh, 2018), osteoporosis (Mizuno et al., 2015), chronic kidney disease (Hata et al., 2021), hematological manifestations like iron deficiency and vitamin B12 (cobalamin) deficiency, hypertension, and atherosclerosis have been linked to *H. pylori* infection (Tsay and Hsu, 2018; Fang et al., 2019; Franceschi et al., 2019; Mansori et al., 2020a; Mansori et al., 2020b).

Involvement of *H. pylori* in this wide array of diseases may be partly related to the fact that this bacterial infection contributes not only to local inflammation but also to systemic inflammation (Tsay and Hsu, 2018; Fang et al., 2019; Franceschi et al., 2019; Mansori et al., 2020a; Mansori et al., 2020b). In turn, the latter can instigate several extra-gastrointestinal disorders such as metabolic syndrome, diabetes mellitus, insulin resistance, and CVD (Tsay and Hsu, 2018; Fang et al., 2019; Franceschi et al., 2019; Mansori et al., 2020a; Mansori et al., 2020b; Kountouras et al., 2021). Of particular interest, recent studies have found that *H. pylori* may be responsible for the initiation, progression, and complications of atherosclerotic plaque formation. Hence, this review was undertaken to critically examine the evidence concerning *H. pylori* chronic infection as a risk factor of atherosclerosis.

## 2 Current evidence

### 2.1 *H. pylori* infection modifies carotid intima-media thickness (CIMT)

One early indication of atherosclerosis is thickening of the carotid artery. In atherosclerosis, this blood vessel narrows, and blood flow becomes compromised thereby increasing the risk of CVD. Indeed, CIMT, which measures the thickness of the inner two layers (the intima and media) of a carotid artery, is an early diagnostic tool of atherosclerosis as this thickening appears even in asymptomatic pre-atherosclerosis patients. Relatedly, several reports support a positive correlation between *H. pylori* infection and an increase of CIMT (Table 1). Indeed, CIMT values were significantly higher in *H. pylori*-infected versus non-infected subjects, and the levels of *H. pylori*-IgG were positively correlated with the increased CIMT measurements (Shan et al., 2018). Similar results were obtained in *H. pylori*-positive men, younger than 50 years of age, who showed a higher incidence of carotid atherosclerosis than non-*H. pylori*-infected counterparts (Zhang et al., 2019). In addition, subjects without carotid atherosclerosis, but with *H. pylori* infection, were found to have higher CIMT values than those free of the infection (Zhang et al., 2019). Importantly, a follow up of subjects with persistent *H. pylori* infection after 5 years revealed a significantly higher incidence of carotid atherosclerosis compared to subjects who were *H. pylori* negative (Zhang et al., 2019). This clearly shows a potentially causative effect of this bacterial infection with atherosclerosis. Indeed, a cross-sectional study shows a positive association between *H. pylori* infection, CIMT and carotid atherosclerosis, independent of classical risk factors (Zhang et al., 2021). In agreement with this, several meta-analyses show that *H. pylori* infection can significantly increase CIMT and lead to subclinical atherosclerosis (Wang et al., 2021b; Shi et al., 2022; Simon et al., 2022).

**TABLE 1:** Major evidence correlating *H. pylori* infection with atherosclerosis.

Evidence	Findings	References
CIMT	Positive correlation between high CIMT values and *H. pylori* infection	Shan et al. (2018), Zhang et al. (2019), Wang et al. (2021b), Talari et al. (2021), Zhang et al. (2021), Shi et al. (2022), Simon et al. (2022)
	Correlation exacerbated when *H. pylori* infection is coupled with certain comorbidities	Bao-Ge et al. (2017), Feng et al. (2018a), Yu et al. (2019), Choi et al. (2022)
Dyslipidemia	H. *pylori* infected subjects have lower levels of HDL and higher levels of LDL.	Lee et al. (2018), Abdu et al. (2020), Kim et al. (2016), Liang et al. (2021), Choi et al. (2019), Medrek-Socha et al. (2018), Shan et al. (2017)
	H. *pylori* eradication therapy increases HDL level and restores LDL/HDL ratio	Iwai et al. (2019), Park et al. (2021)
Systemic immune response	Chronic inflammation caused by *H. pylori* infection can generate persistent oxidative stress and modify LDL into oxidized-LDL.	Hansson et al. (2006), Sies et al. (2017), Butcher et al. (2017), Gao and Liu (2017), Luo et al. (2017), Krupa et al. (2021)
	Serum Ox-LDL and 8-OHdG levels are higher in T2DM patients with *H. pylori* infection	Nasif et al. (2016)
	Potential association between *H. pylori*, the inflammatory cytokine YKL-40, and atherosclerosis	Cooke et al. (2001), Wu et al. (2004), Martinet et al. (2002), Rathcke and Vestergaard (2006), Xu et al. (2016)
Vitamin B12 deficiency	*H. pylori*-induced atrophic gastritis and reduction in the levels of intrinsic factor protein lead to Vitamin B12 deficiency	Supiano (1987), Kaptan et al. (2000), Carmel et al. (2001), Dierkes et al. (2003), Sipponen et al. (2003), Soyocak et al. (2021)
	*H. pylori*–induced vitamin B12 deficiency is suggested to play a key role in atherosclerosis via several mechanisms one of which is inhibition of the enzyme methionine synthase leading to high production of homocysteine. Hyperhomocysteinemia is implicated in atherosclerosis via several mechanisms	Zhang et al. (2000), Zhang et al. (2009), Steed and Tyagi (2011), Kozlowski et al. (2012), Zhao et al. (2017), Wu et al. (2019)
CagA	CagA is released systemically from CagA-injected gastric epithelial cells via exosomes. At the vascular endothelium, the virulence factor can stimulate NF-κB and STAT3 in endothelial cells and promote the mounting of an immune reaction at the atherosclerotic plaques by interaction with anti-CagA antibodies	Ansari and Yamaoka (2020), Tegtmeyer et al. (2017), Shimoda et al. (2016), Tahmina et al. (2022), Qiang et al. (2022)
	CagA+ exosomes cause endothelial dysfunction	Guo et al. (2021), Xia et al. (2022)
	When taken up by macrophages in plaques, CagA accelerates foam cells formation	Yang et al. (2019)
	CagA promotes platelets aggregation	Byrne et al. (2003); Riad (2021)

Abbreviations: CIMT, carotid intima-media thickness test; Ox-LDL, oxidized low-density lipoprotein; 8-OHdG, 8-hydroxy-2′-deoxyguanosine, T2DM, Type 2 diabetes mellitus.

One of the key interplayers in the potential association between atherosclerosis and *H. pylori* infection is the cytotoxin-associated gene A (CagA). Indeed, studies involving both the right and left coronary artery of patients who underwent an upper GI endoscopy show higher CIMT values among patients infected with a *H. pylori* strain positive for CagA, compared to patients infected with CagA-negative *H. pylori* (Talari et al., 2021). The CagA-positive group also exhibited higher levels of high-sensitivity C-reactive protein (hsCRP), a marker of elevated inflammatory response (Talari et al., 2021). These studies led to the conclusion that CagA+ *H. pylori* strain can induce a systemic inflammatory response that may contribute to the development of atherosclerosis (Talari et al., 2021). Notably, changes in CIMT values were found to be even more prominent when *H. pylori* infection was coupled with certain comorbidities. For instance, CIMT values were highest among subjects with an *H. pylori* infection and alcoholic liver disease (ALD), compared to other groups with only one or neither of the conditions (Bao-Ge et al., 2017). Additionally, the coexistence of *H. pylori* infection and early-stage diabetic kidney disease (DKD) causes a significant increase in CIMT measurements, and potentiates the risk of developing atherosclerosis in type 2 diabetic patients (Feng et al., 2018a). In confirmation, two recent studies revealed that patients with both *H. pylori* infection and nonalcoholic fatty liver disease (NAFLD) exhibited the highest risk of carotid artery plaque formation and arterial stiffness (Yu et al., 2019; Choi et al., 2022). Taken together, these findings support the notion that *H. pylori* infection is associated with higher CIMT measurements, suggesting a correlation with the eventual incidence of atherosclerosis, in healthy individuals or ones with other co-morbidities.

### 2.2 *H. pylori* infection precipitates dyslipidemia

Maintaining appropriate blood lipid profiles is critical for the prevention of atherosclerotic plaque buildup. Among serum lipids, cholesterol exhibits a high tendency to accumulate on vascular walls, potentially narrowing the lumen and obstructing blood flow. Interestingly, one of the major mechanisms by which *H. pylori* could precipitate the progression of atherosclerosis is by modifying serum lipid levels and profiles (Table 1) (Vijayvergiya and Vadivelu, 2015).

Current evidence suggests that *H. pylori* is responsible for an impairment in lipid metabolism (Adachi et al., 2018). A chronic infection with *H. pylori* can modify host body lipid distribution by activating pro-inflammatory factors, decreasing lipolysis, and enhancing *de novo* synthesis of fatty acids in the liver (Wang et al., 2022). *H. pylori* can also directly act on the liver to modify body lipid profiles by inducing liver dysfunction and elevating small intestinal mucosal permeability, facilitating the invasion of bacterial endotoxins to the liver through the portal vein, causing hepatic tissue damage (Wang et al., 2022). Importantly, *H. pylori* was reported as an independent risk factor for impaired lipid profiles, manifested as reduced high-density lipoprotein (HDL) and elevated low-density lipoprotein (LDL) levels (Buzás, 2014; Zhao et al., 2019; Hashim et al., 2022; Wang et al., 2022). It is not surprising then that dyslipidemia is prevalent in *H. pylori*-suspected patients where 87.2% of *H. pylori* positive subjects had at least one abnormality in lipid profile (Abdu et al., 2020). This argument is cemented by the finding that *H. pylori*-triggered deterioration in lipid metabolism or dyslipidemia is alleviated when this bacterium is eradicated (Park et al., 2021; Wang et al., 2022).

A recent study aiming to evaluate whether current *H. pylori* infection, detected using a rapid urease test [Campylobacter-like organism test (CLO)], is correlated with subclinical atherosclerosis showed that CLO-positive subjects are more likely to have significant coronary artery stenosis compared to CLO-negative subjects (Lee et al., 2018). The CLO-positive subjects also showed lower levels of HDL-cholesterol and higher mean levels of triglycerides compared to the CLO-negative subjects (Lee et al., 2018). The risk of having a coronary stenosis was even more prominent after adjusting for age, sex, and other factors that influence coronary artery stenosis, such as systolic blood pressure (BP), fasting glucose, HDL-cholesterol, anti-hypertension/diabetic medications, lipid-lowering agents, and antiplatelet agents (Lee et al., 2018). A recent cross-sectional study showed that *H. pylori*-infected patients exhibit higher LDL, triglycerides and cholesterol levels than control patients’ (Nigatie et al., 2022). This is further supported by other findings showing that *H. pylori* seropositivity is a significant risk factor for higher levels of LDL-cholesterol, triglycerides, BMI and lower levels of HDL-cholesterol, further establishing a role for *H. pylori* infection in dyslipidemia (Table 1) (Kim et al., 2016).

In asymptomatic healthy individuals, arterial stiffness, LDL-cholesterol levels, and the prevalence of dyslipidemia were significantly higher in the *H. pylori*-seropositive group compared to the *H. pylori*-seronegative group (Choi et al., 2019). In confirmation, findings from a recent study where participants were divided into three groups (healthy non-*H. pylori* infected, and symptomatic and asymptomatic *H. pylori* infected individuals), indicated that cholesterol levels were significantly higher in the symptomatic group than the asymptomatic and healthy groups (Medrek-Socha et al., 2018). Relevantly, both of the infected groups showed higher LDL and lower HDL concentrations than the healthy group (Medrek-Socha et al., 2018). Other supporting data include the finding that subjects infected by *H. pylori*, specifically males between 55 and 74 years of age, had significantly higher levels of LDL-cholesterol (Shan et al., 2017). A recent observational study in chronic gastritis patients showed that *H. pylori* eradication therapy causes a significant increase in HDL levels and a significant decrease of LDL/HDL ratio, a measure of the risk of atherosclerosis (Iwai et al., 2019). Taken together, it is becoming increasingly evident that infection with *H. pylori* is associated with dyslipidemia, a key contributing factor to atherosclerotic plaque formation.

### 2.3 *H. pylori* infection can induce systemic immune responses

Key parameters implicated in systemic inflammation are adhesion molecules and pro-inflammatory cytokines. In this context, it has been reported that *H. pylori* can induce upregulation of adhesion molecules on gastric epithelium as well as promote the release of several cytokines like IL-1, IL-6 and TNF -ɑ, which then activate leukocytes and precipitate systemic inflammation (Vijayvergiya and Vadivelu, 2015). This *H. pylori*-induced chronic inflammation can precipitate persistent oxidative stress, with various noxious effects such as DNA damage, mitochondrial membrane damage and pro-inflammatory immune responses (Butcher et al., 2017; Sies et al., 2017). This contributes to a wide range of extra-gastrointestinal tract abnormalities, including atherosclerosis.

**FIGURE 1:**
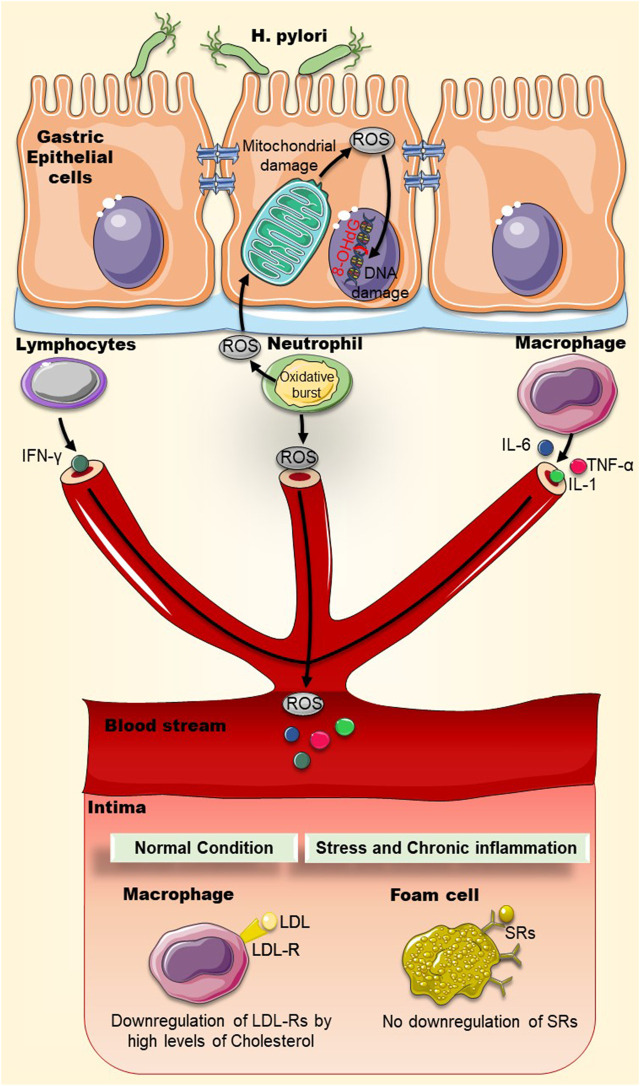
Chronic inflammation and oxidative stress may increase the uptake of oxidized-LDL particles by scavenger receptors on macrophages. Chronic *H. pylori* infection can cause local and systemic inflammation that may accelerate atherosclerotic plaque formation. Oxidative stress (ROS) and inflammatory markers (IL-1, IL-6, TNF-α, IFN-γ) generated by immune cells and mitochondrial membrane disruption can damage DNA where deoxyguanosine becomes oxidized into 8-OHdG. LDL particles are modified by oxidative stress into oxidized-LDL (Ox-LDL). Ox-LDL particles do not bind to LDL-Rs but rather bind to scavenger receptors (SRs), present on macrophages. Unlike LDL-Rs, SRs are not downregulated by high levels of cholesterol and accumulate more cholesterol and fats into macrophages, causing the eventual conversion of macrophages into foam cells. Abbreviations: LDL: Low-density lipoprotein; Ox-LDL: Oxidized low-density lipoprotein; LDL-R: low-density lipoprotein receptor; ROS: Reactive oxygen species; SRs: Scavenger receptors; 8-OHdG: 8-hydroxy-2′-deoxyguanosine.

Under normal oxidative conditions, LDL particles are taken up by macrophages via receptor-mediated endocytosis by the LDL-Receptors (LDL-Rs). LDL-Rs can be downregulated by elevated levels of cholesterol, limiting the uptake of fat. However, LDL particles are extremely sensitive to oxidative damage and can be modified into oxidized-LDL (Ox-LDL) during chronic *H. pylori* infection, for instance. Ox-LDL are atherogenic because they do not bind to LDL-Rs but have the capacity to bind and activate a group of receptors collectively known as scavenger receptors (SRs). These SRs are present on macrophages, but unlike LDL-Rs they are not downregulated by high levels of cholesterol, and can keep on accumulating cholesterol and fats, eventually causing the conversion of macrophages into foam cells, a hallmark of atherosclerosis (Figure 1) (Gao and Liu, 2017; Luo et al., 2017). When lipoproteins accumulate in the intima, the endothelium becomes poised to secrete a repertoire of chemokines and display several leukocyte adhesion molecules, favoring the recruitment of immune cells. An immune response is then mounted, leading to the accumulation of pro-inflammatory cytokines, proteases, and vasoactive molecules. This local inflammation can exacerbate plaque growth in blood vessels, furthering their obstruction (Hansson et al., 2006).

Ox-LDL levels were also found to be higher in serum of *H. pylori*–infected type II diabetes mellitus (T2DM) patients than in serum of diabetic patients without *H. pylori* infection or non-diabetics (Table 1) (Nasif et al., 2016). More, the role of *H. pylori* infection in accelerating foam cell formation in animals with high-fat diet has also been reported (Krupa et al., 2021). In these animals, a dramatic increase in systemic inflammatory markers fueling a pro-atherogenic endothelial cell environment was noted. Infiltration of inflammatory cells, elevation of oxidative stress, formation of foam cells, and presence of pro-atherogenic molecules like 7-ketocholesterol and aldehydes were prominently evident (Krupa et al., 2021).

Oxidative damage also impacts both nuclear and mitochondrial DNA. For instance, deoxyguanosine becomes oxidized into 8-hydroxy-2′-deoxyguanosine (8-OHdG) (Cooke et al., 2001). Indeed, this oxidized DNA molecule is often used as a marker of oxidative damage. Moreover, it is reportedly found-in rather excessive amounts-in atherosclerotic plaques (Wu et al., 2004), inside macrophages, smooth muscle cells, and endothelial cells, demonstrating strong oxidative DNA damage and repair (Martinet et al., 2002). Likewise, levels of 8-OHdG and serum Ox-LDL levels in T2DM patients appear to be directly associated with *H. pylori* infection (Nasif et al., 2016). This lends support to the notion that *H. pylori* infection contributes to the pathogenesis of atherosclerosis by virtue of increasing serum Ox-LDL and 8-OHdG (Figure 1).

An interesting relation between vascular dementia, inflammation and atherosclerosis is emerging. Indeed, *H. pylori*-positive patients with vascular dementia (VD) had greater CIMT values and higher levels of YKL-40 cytokine [a biomarker of inflammation (Rathcke and Vestergaard, 2006)] than *H. pylori*-negative VD patients (Xu et al., 2016). Additionally, CIMT was positively correlated with serum levels of YKL-40 cytokine independent of traditional atherosclerotic risk factors (Xu et al., 2016). This finding suggests a potential association between *H. pylori*, YKL-40 cytokines and atherosclerosis, warranting further investigation (Xu et al., 2016). Overall, *H. pylori* infection-induced inflammation may be able to precipitate the development of atherosclerosis as summarized in Table 1.

### 2.4 *H. pylori* infection can cause vitamin B12 deficiency

Atrophic gastritis is chronic inflammation of the gastric mucosa that can result in stomach atrophy along with low or absent gastric acid secretion and inadequate production of intrinsic factor (IF). This factor is a protein that binds and facilitates the transit of vitamin B12 (cobalamin) through the small intestine to be absorbed into the bloodstream (Hall and Appelman, 2019). In the absence of gastric secretions or IF, the absorption of food-bound vitamin B12 is impaired (Neumann et al., 2013). As a result, people suffering from chronic atrophic gastritis have been documented to suffer from cobalamin deficiency (Allen, 2008). Of particular interest to this review, an untreated *H. pylori* infection can eventually cause atrophic gastritis, implicating *H. pylori* in vitamin B12 deficiency (Supiano, 1987). This causality was investigated in several studies, with the conclusion that there is a probable association between cobalamin deficiency and *H. pylori* infection (Kaptan et al., 2000; Carmel et al., 2001; Dierkes et al., 2003; Sipponen et al., 2003; Raut and Chandel, 2014; Civan, 2020; Soyocak et al., 2021). These findings have potential implications to atherosclerosis because vitamin B12 malabsorption can promote the progression of atherosclerosis (Zhao et al., 2017). In fact, cobalamin is a cofactor for the enzyme methionine synthase catalyzing the conversion of homocysteine to methionine (Kozlowski et al., 2012). In the case of vitamin B12 deficiency, methionine synthase is non-functional, escalating homocysteine levels (Sipponen et al., 2003). A recent study comparing homocysteine levels of *H. pylori* positive subjects to those of *H. pylori* negative individuals found that homocysteine levels were significantly higher in the infected subjects (Huang et al., 2022).

**FIGURE 2:**
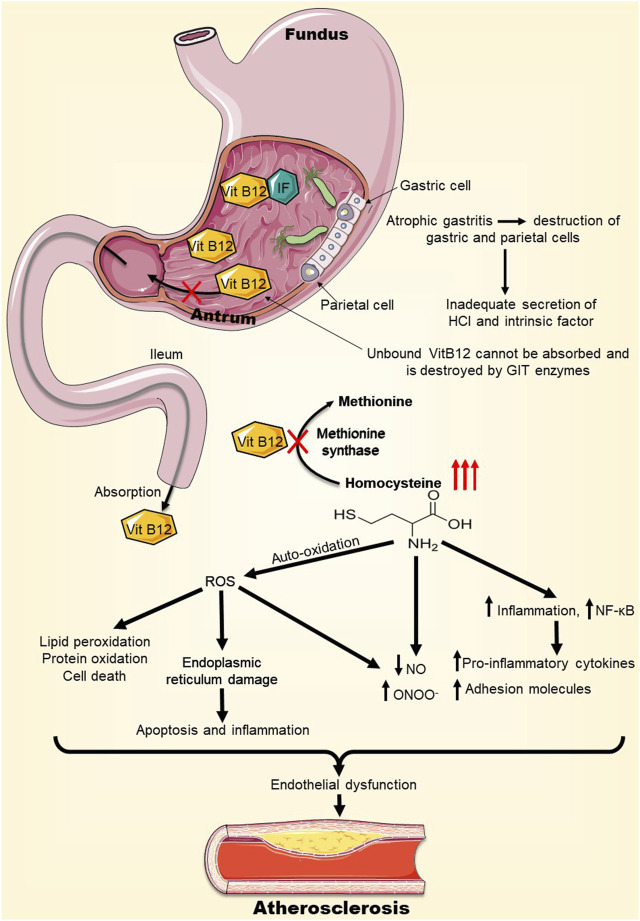
*H. pylori*-induced Vitamin B12 deficiency may precipitate atherosclerosis. *H. pylori* infection can lead to atrophic gastritis and destruction of parietal cells, which secrete intrinsic factor (IF), essential for vitamin B12 absorption. As a result, *H. pylori* infection can cause vitamin B12 deficiency. Vitamin B12 deficiency inhibits the enzyme methionine synthase, escalating homocysteine levels. High levels of homocysteine in the vasculature can lead to endoplasmic reticulum stress, oxidative stress, and inflammation, overall causing endothelial dysfunction which promotes atherosclerosis. Abbreviations: IF, Intrinsic Factor; ROS, Reactive oxygen species; NO, Nitric oxide; ONOO^−^, Peroxynitrite; NF-kB, Nuclear factor Kappa B.

Hyperhomocysteinemia (HHcy) is suggested to play a key role in atherosclerosis via several mechanisms (Zhao et al., 2017). The auto-oxidation of homocysteine is a source of reactive oxygen species (ROS), which at high levels in the vasculature can cause lipid peroxidation, protein oxidation, and even cell death, eventually leading to vascular injuries (Steed and Tyagi, 2011). HHcy by itself as well as HHcy-generated ROS can induce peroxynitrite (ONOO^−^) formation, effectively reducing nitric oxide (NO) bioavailability in the vasculature. Generation of ONOO^−^ and the decrease of NO levels can cause dysfunction of the vascular endothelium, instigating atherosclerosis and other diseases of the vasculature (Zhang et al., 2000). HHcy-induced oxidative stress can as well affect the endoplasmic reticulum of endothelial cells, thereby inducing apoptosis and inflammation, and dysregulation of lipid metabolism (Wu et al., 2019). Furthermore, elevated levels of homocysteine can induce the activation of nuclear factor kappa B (NF-κB) transcription factor which activates the expression of different cytokines, chemokines, and leukocyte adhesion molecules; important mediators of vascular inflammation, pro-thrombotic state, and atherogenesis (Figure 2) (Cattaneo, 1999; Zhang et al., 2009; Riad, 2021). Taken together, it is becoming more evident that *H. pylori-*induced vitamin B12 malabsorption can lead to elevated levels of homocysteine, which may then promote the development of atherosclerosis by various mechanisms (Figure 2).

### 2.5 The role of CagA in atherosclerosis

**FIGURE 3:**
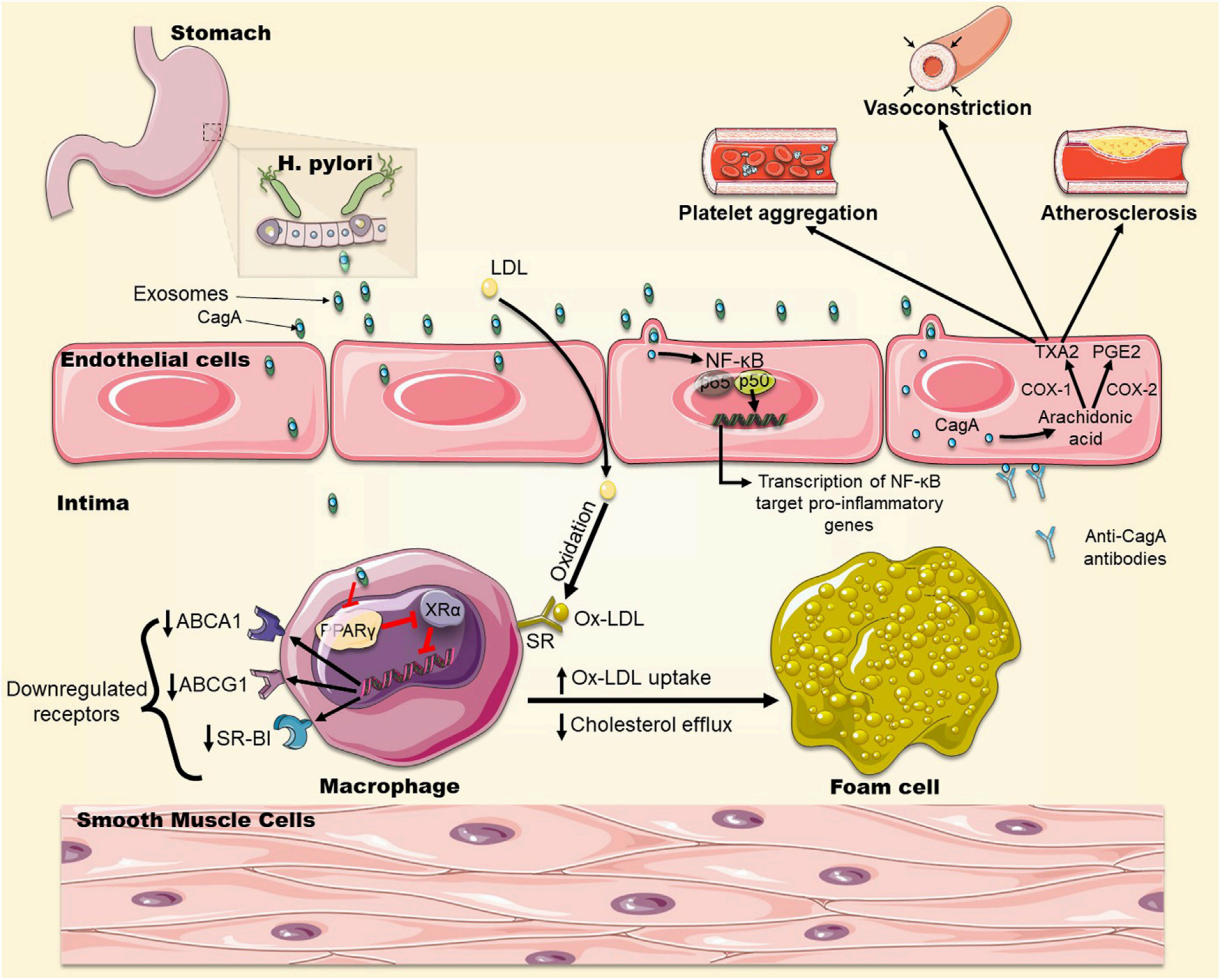
The role of CagA in foam cell formation. CagA is released in exosomes from *H. pylori*-infected gastric cells and becomes engulfed by macrophages in the intima of arteries. CagA downregulates the expression of transcription factors PPARγ and LXRα, which control the expression of the cholesterol efflux transporters ABCA1, ABCG1 and SR-BI on the surface of macrophages leading to cholesterol accumulation in macrophages and promoting foam cell formation. In addition, OxLDL infiltrates macrophages via SRs which are not downregulated by cholesterol, exacerbating foam cell formation. CagA also stimulates NF-kB signaling leading to the upregulation of expression of pro-inflammatory genes. CagA also elevates the levels of COX-1 and COX-2, leading to a higher production of TXA2 and PGE2, promoting platelets aggregation and vasoconstriction. CagA can be expressed on cells of the atherosclerotic plaque, to which anti-CagA antibodies will bind, exacerbating immune reactions.

CagA, a virulence factor protein expressed by certain strains of *H. pylori,* is responsible for the development of gastric diseases and is suspected to be involved in extra-gastric diseases (Ansari and Yamaoka, 2020)*.* During infection, *H. pylori* injects this cytotoxin into host gastric cells, through a Type IV secretion system, where CagA can modify host cell signaling pathways (Tegtmeyer et al., 2017). *H. pylori*-infected gastric epithelial cells were found to release extracellular vesicles (exosomes) containing CagA. These exosomes can be delivered, through the bloodstream, to vascular endothelia distant from the primary site of infection (Table 1) (Shimoda et al., 2016). This finding may be one way to explain the systemic clinical effects thought to be mediated by *H. pylori*, including atherosclerosis. Indeed, these circulating CagA-containing exosomes may adhere to distant endothelial monolayers and deliver the CagA protein into endothelial cells, where it can stimulate NF-κB signaling. This signaling may lead to a chronic inflammatory condition, endothelial cell dysfunction, and consequently plaque formation. Similarly, Wang *et al.* reported that extracellular vesicle (outer membrane vesicles) derived from CagA^+^ *H.pylori*-infected cells accelerated atherosclerotic plaque formation in ApoE^−/−^ mice via NF-κB signaling (Figure 3) (Wang et al., 2021a). Exosomes-delivered CagA to endothelial cells were also found to activate the pro-inflammatory transcription factor STAT3 (Tahmina et al., 2022). In another mechanism, CagA-containing exosomes may directly fuse with cells of the atherosclerotic plaques, exposing CagA on the plaque surface to be recognized by anti-CagA antibodies, leading to immune reactions at the atherosclerotic plaque (Figure 3) (Shimoda et al., 2016).

PMSS1 is a CagA-positive *H. pylori* strain which can secrete the CagA virulence factor into host cells. PMSS1 *H. pylori*-infected mice have shown accelerated growth of atherosclerotic plaques that was dependent on dietary risk factors and genetic susceptibility (Yang et al., 2019). Immunohistochemical analysis of the vasculature of these mice revealed that the atherosclerotic plaques contained macrophage-derived foam cells. These cells significantly accumulate neutral lipid in the presence of PMSS1 infection, suggesting that the bacteria may help in the formation of foam cells (Yang et al., 2019).

This notion was reinforced by other *in vitro* and *in vivo* studies. Exosomes derived from *H. pylori*-infected gastric epithelial cells (Hp-GEC-EVs), were able to induce conversion of macrophages into foam cells when taken up by macrophages. Interestingly, CagA present in Hp-GEC-EVs would end up in the atherosclerotic plaques leading to exacerbation of the obstructive inflammatory process (Yang et al., 2019). It was also found that CagA mediated the formation of foam cells through downregulation of the expression of transcription factors PPARγ and LXRα, which control the expression of the cholesterol efflux transporters ABCA1, ABCG1 and SR-BI on the surface of macrophages, leading to cholesterol accumulation in macrophages and promoting foam cell formation (Figure 3) (Yang et al., 2019). A recent study comparing the virulence of CagA^+^ *H. pylori* to CagA^−^ *H. pylori* in mice found that the former strain colonized gastric mucosa more effectively, causing endothelial dysfunction and enhancing atherosclerosis through ROS production, which was induced by CagA^+^ exosomes (Xia et al., 2022). Another study demonstrated that *H. pylori* infection was an independent risk factor for intracranial atherosclerosis, especially in women younger than 60 years of age, and that CagA^+^ exosomes significantly limited brain endothelial functioning *in vitro* (Guo et al., 2021). As well, a meta-analysis showed that *H. pylori* infection can significantly increase CIMT, especially in CagA^+^ individuals (Shi et al., 2022).

CagA-positive *H. pylori* strains can also promote platelet aggregation. Indeed, CagA is suspected to increase cyclooxygenase-1 (COX-1) and cyclooxygenase-2 (COX-2) production in the vascular endothelium resulting in higher thromboxane (TXA2) and prostaglandins levels, both of which are known to modify platelet aggregation and contribute to atherosclerosis (Figure 3) (Byrne et al., 2003; Riad, 2021).

Hence, CagA^+^ *H. pylori* infection may accelerate atherosclerotic plaque formation via several CagA-dependent mechanisms: CagA packaged in exosomes can modify macrophage intracellular cholesterol levels by increasing the import of cholesterol or decreasing cholesterol efflux which facilitates foam cell formation. Otherwise, CagA can promote the development of atherosclerotic plaques via the stimulation of NF-κB, platelet aggregation, and the interaction with anti-CagA antibodies (Figure 3).

## 3 The counter-evidence

Despite the multitude of studies demonstrating a positive association between *H. pylori* infection and atherosclerosis, some studies report otherwise (Ahmadnia et al., 2013; Jukic et al., 2017; Feng et al., 2018b; Wernly et al., 2022). A recent cross-sectional study aiming to examine the relationship between *H. pylori* infection and the severity of coronary atherosclerosis included patients with coronary artery disease (CAD) and who underwent coronary artery bypass grafting surgery. Coronary angiograms were scored by quantitative assessment based on three angiographic parameters: the vessel score, Gensini score, and angiographic severity score. The results showed that hypertension, systolic pressure, diastolic pressure, and total cholesterol values were higher in *H. pylori*-positive subjects compared to *H. pylori*-negative subjects. HDL-cholesterol values, on the other hand, were markedly lower in the *H. pylori*-positive than in the negative subjects. However, no significant differences were found in vessel score, Gensini score and angiographic severity score, or in the use of medications that affect atherogenesis, concluding that the pathogenesis of stable chronic CAD is mainly caused by traditional risk factors rather than the influence of *H*. *pylori* infection (Jukic et al., 2017). Furthermore, a cross-sectional investigating the correlation between the average of CIMT values and *H. pylori* infection did not report significant differences in CIMT measurements between *H. pylori* positive and negative subjects (Feng et al., 2018b). However, traditional atherosclerosis risk factors (like gender, BMI, waistline, lipid profiles) did correlate with significant differences in CIMT scores suggesting no correlation between *H. pylori* infection and early atherosclerosis (Feng et al., 2018b). Moreover, the presence of *H. pylori* in atherosclerotic plaques was investigated in the iliac arteries of 25 patients with end stage renal disease (ESRD) who were undergoing kidney transplantation. Although atherosclerotic plaques were present in 21 patients (84%), *H. pylori* was not detected by polymerase chain reaction and no significant relationship between atherosclerosis and gastric *H. pylori* infection could be established (Ahmadnia et al., 2013). Moreover, despite reporting an independent risk between *H. pylori* infection and CVD, the difference did not translate into CVD mortality between patients with and without the bacteria in a cohort (Wernly et al., 2022). Hence, conflicting data exist regarding the relationship between *H. pylori* and atherosclerosis and further investigations are needed in order to elucidate this relationship.

## 4 Conclusion

**FIGURE 4:**
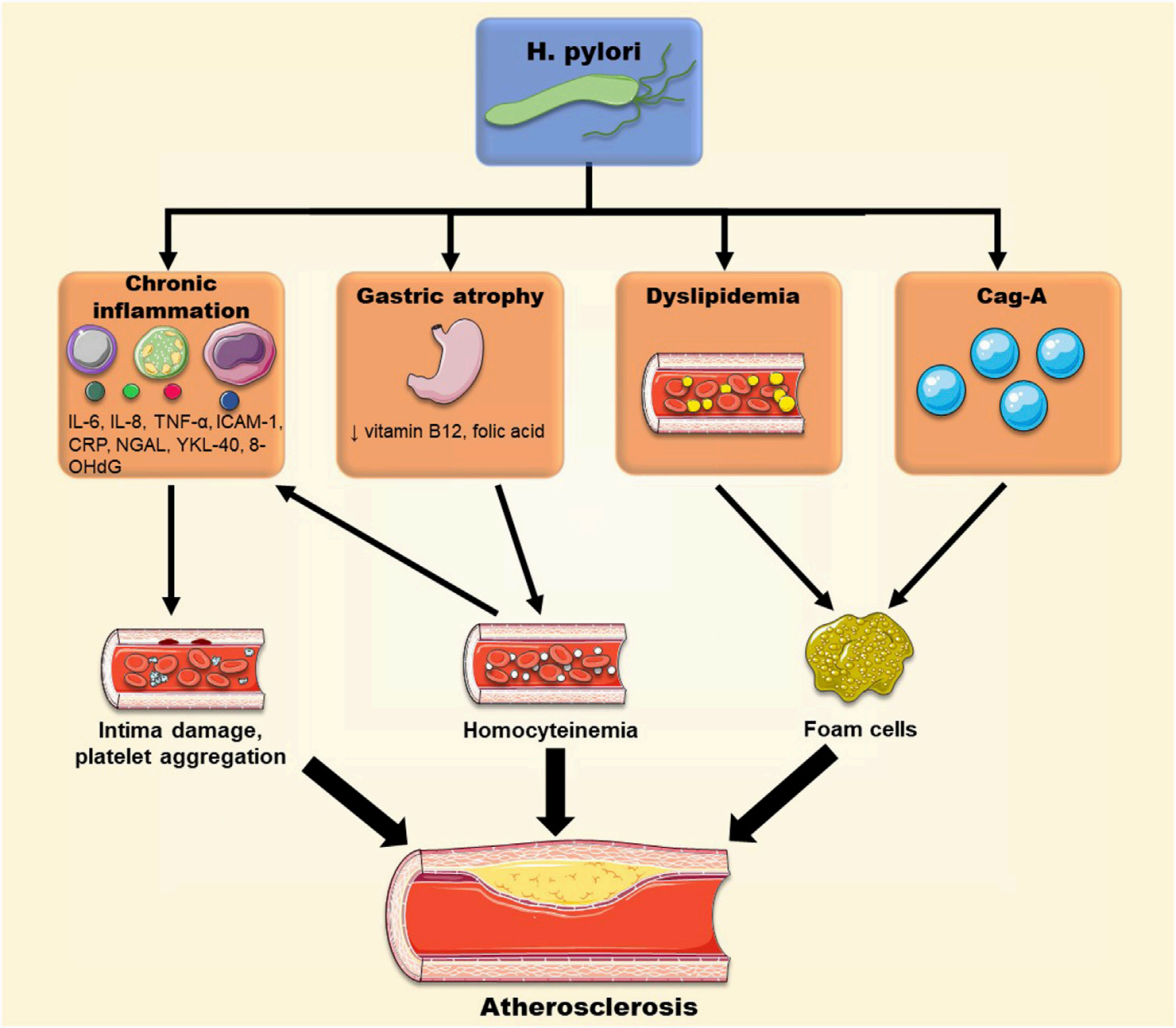
Different pathways through which *H. pylori* may promote atherosclerotic plaque formation. *H. pylori* infection can have systemic effects and cause chronic inflammation, dyslipidemia, Vitamin B12 deficiency and homocysteinemia, and promote foam cell formation, contributing to the progression of atherosclerosis.

The extra-gastrointestinal manifestations of *H. pylori* infection have linked this pathogen to the progression of atherosclerosis. In addition, the bacteria may account for the development of atherosclerotic plaques through several mechanisms including dyslipidemia, systemic inflammation, oxidative stress and acceleration of foam cell formation. When different strains of the bacteria were compared, *H. pylori* strains positive for CagA showed a higher correlation with the incidence and pathogenesis of atherosclerosis, through various mechanisms involving exosomes, for example, (Figure 4). As such, interventions to eradicate *H. pylori* may help in the reduction of the prevalence of atherosclerosis. Paradoxically, some studies could not establish a role for *H. pylori* in atherosclerosis (Ahmadnia et al., 2013; Jukic et al., 2017; Feng et al., 2018b). The contradiction may be related to the presence of confounding variables, such as the methods used for diagnosis of *H. pylori* diagnostic (serology vs. rapid urease test), selection bias of geographic regions, or the study of different *H. pylori* strains, like CagA-positive or CagA-negative strains. In conclusion, *H. pylori* may exacerbate atherosclerosis, but further supporting evidence is warranted to place it alongside the traditional risk factors of atherosclerosis.

## Abbreviations

Cag-A, Cytotoxin-Associated Gene; IL-6, Interleukin 6; IL-8, Interleukin 8; TNF-α, Tumor necrosis factor α; ICAM-1, Intercellular adhesion molecule 1; CRP, C-Reactive Protein; NGAL, neutrophil gelatinase-associated lipocalin; 8-OHdG, 8-hydroxy-2′-deoxyguanosine.
